# Social isolation and psychosis: an investigation of social interactions and paranoia in daily life

**DOI:** 10.1007/s00406-021-01278-4

**Published:** 2021-06-15

**Authors:** Anne-Kathrin J. Fett, Esther Hanssen, Marlie Eemers, Emmanuelle Peters, Sukhi S. Shergill

**Affiliations:** 1 Department of Psychology, City, University of London, London, ECIV 0HB UK; 2 Department of Psychosis Studies, King’s College London, Institute of Psychiatry, Psychology and Neuroscience, De Crespigny Park, London, SE5 8AF UK; 3 Department of Developmental and Clinical Psychology, Faculty of Behavioural and Movement Sciences, VU Amsterdam, Van der Boechorststraat 1s, 1081 BT Amsterdam, The Netherlands; 4 Department of Psychology, King’s College London, Institute of Psychiatry, Psychology and Neuroscience, De Crespigny Park, London, SE5 8AF UK; 5 South London and Maudsley NHS Foundation Trust, Bethlem Royal Hospital, Monks Orchard Road, Beckenham, Kent, BR3 3BX UK

**Keywords:** Social functioning, Paranoid delusions, Psychosis continuum, Experience sampling, Non-affective psychosis

## Abstract

Social isolation has been suggested to foster paranoia. Here we investigate whether social company (i.e., being alone vs. not) and its nature (i.e., stranger/distant vs. familiar other) affects paranoia differently depending on psychosis risk. Social interactions and paranoid thinking in daily life were investigated in 29 patients with clinically stable non-affective psychotic disorders, 20 first-degree relatives, and 26 controls (*n* = 75), using the experience sampling method (ESM). ESM was completed up to ten times daily for 1 week. Patients experienced marginally greater paranoia than relatives [*b* = 0.47, *p* = 0.08, 95% CI (− 0.06, 1.0)] and significantly greater paranoia than controls [*b* = 0.55, *p* = 0.03, 95% CI (0.5, 1.0)], but controls and relatives did not differ [*b* = 0.07, *p* = 0.78, 95% CI (− 0.47, 0.61)]. Patients were more often alone [68.5% vs. 44.8% and 56.2%, respectively, *p* = 0.057] and experienced greater paranoia when alone than when in company [*b* = 0.11, *p* = 0.016, 95% CI (0.02, 0.19)]. In relatives this was reversed [*b* = − 0.17, *p* < 0.001, 95% CI (− 0.28, − 0.07)] and in controls non-significant [*b* = − 0.02, *p* = 0.67, 95% CI (− 0.09, 0.06)]. The time-lagged association between being in social company and subsequent paranoia was non-significant and paranoia did not predict the likelihood of being in social company over time (both *p*’s = 0.68). All groups experienced greater paranoia in company of strangers/distant others than familiar others [*X*^2^(2) = 4.56, *p* = 0.03] and being with familiar others was associated with lower paranoia over time [*X*^2^(2) = 4.9, *p* = 0.03]. Patients are frequently alone. Importantly, social company appears to limit their paranoia, particularly when being with familiar people. The findings stress the importance of interventions that foster social engagement and ties with family and friends.

## Introduction

Individuals with non-affective psychosis frequently experience chronic social isolation [1, 2]. Paranoid delusions, the fixed false beliefs that others have harmful intentions towards oneself, are one of the key symptoms of the disorder and have been suggested to contribute to this social withdrawal from others [3, 4]. Being with others may increase paranoid delusions through social anxiety or feelings of social threat, particularly in those with a psychotic disorder or an increased vulnerability for psychosis [5]. If paranoid delusions are elevated in the company of others, the individual could reduce social contact as a safety behaviour to prevent the perceived threat from occurring. If perceived threat and distress subsequently decrease, social withdrawal is reinforced [3]. Importantly, while such safety behaviour might be effective in the short term, a socially isolated person is unable to revise paranoid thoughts on the basis of positive social interactions [6]. Thus, in absence of counterevidence that disconfirms paranoid thinking, ideas of threat and paranoia may flourish [3, 7]. Social isolation may therefore work to maintain or to aggravate paranoid delusions, which could ultimately lead to a self-perpetuating cycle of social exclusion [8].

It is clear that paranoia fluctuates in intensity throughout the day [9, 10]. Changes in social surroundings (e.g., the presence of others) may be crucial in determining these short-term fluctuations, conceptualized as ‘momentary’ paranoia. Collip et al. [9] used the experience sampling method (ESM), a structured diary technique, to assess the associations between social context and paranoia in a sample comprising acutely paranoid patients, acutely ill but non-paranoid patients, remitted psychotic patients, high-schizotypy participants, and controls. Participants were divided into groups with low, medium, and high trait paranoia, referring to paranoia as a stable phenotype in the general population. Those in the low and medium trait paranoia groups reported greater paranoia in the company of less familiar than more familiar people. This seems intuitively adaptive since one has to be warier of the intentions of a stranger than a familiar individual with whom one has a history of trust. Individuals with high trait paranoia reported greater momentary paranoia than individuals in the low and medium paranoia groups, but their levels of paranoia did not differ when they were in less familiar or familiar company. This suggests that in highly (trait) paranoid individuals momentary paranoia is independent of the social context [9].

However, the study failed to differentiate paranoia levels between the different groups; healthy controls comprised only 56% of the low paranoia group, ‘acutely paranoid patients’ formed only 74% of the high paranoia group and the groups did not differ in terms of their real-life social engagement either. Myin-Germeys et al. [10] examined the effect of social context on delusions using ESM in patients with a diagnosis in the schizophrenia spectrum. The study found that in patients being with familiar others actually decreased the risk of experiencing delusions within 90 min later, while no such effect was present when interacting with strangers [10]. This study supports the premise that interactions with caring and helpful others have a protective effect that can reduce or prevent delusions in patients [7]. Together the two studies suggest that the reactivity to the nature social company might differ as a function of the individual severity of paranoid delusions and psychosis risk [11]. However, the differences in findings could also be due to methodological differences, such as the contemporaneously vs. time-lagged measurement of paranoia [9, 10].

In this study, we aimed to investigate the relationship between social company (i.e., being alone vs. not), its nature (i.e., being with less familiar and familiar others) and paranoia across the psychosis continuum. We included individuals with a diagnosis of non-affective psychosis, controls without a family history of psychosis, and for the first time healthy first-degree relatives with a family history of psychosis. Any associations between social context and paranoid thinking in relatives are unconfounded by factors that are secondary to the clinical disorder, such as stigma or antipsychotic medication use. Thus, including relatives can help to identify whether specific mechanisms are associated with the familial vulnerability to psychosis.

We used ESM to investigate the association between social interactions and paranoid thinking in daily life. We hypothesized that: (1) patients would be alone more often and generally would experience greater paranoia than controls, and that relatives would occupy an intermediate position; (2) in patients paranoia would be greater when alone than when in social company; (3) in patients being alone would predict greater paranoia over time and that reversely greater paranoia would be associated with a higher likelihood of being alone over time (in line with a hypothesized safety mechanism); (4) all groups would show less paranoia in the company of familiar compared to less familiar others; (5) in patients being with familiar but not less familiar others would be associated with lower levels of paranoia over time.

## Methods

### Participants

Eighty-two participants were enrolled in three groups: individuals with a diagnosis of a non-affective psychotic disorder, healthy first-degree relatives of individuals with non-affective psychosis, and controls without a personal or family history of psychosis. Inclusion criteria for all participants were: age between 18 and 65, sufficient command of the English language to understand the ESM application and testing material, estimated Intelligence Quotient > 70. Additional inclusion criteria for patients were: a diagnosis of a non-affective psychosis according to ICD-10 criteria [12], stable on their current pharmacological treatment (> 6 weeks). Exclusion criteria for all participants were: a history of any neurological condition and a diagnosis of alcohol/drug dependence within 6 months prior to study screening. The patient group was recruited via the South London and Maudsley NHS Foundation Trust, the ‘Consent for Consent c4c’ initiative, the OXLEAS, NELFT and SEPT NHS Foundation Trusts in cooperation with the Mental Health Research Network and via other research projects within the Psychosis Studies Department at the Institute of Psychiatry, Psychology, Neuroscience (IoPPN), King’s College London. Relatives were recruited via the mental health charities Mind and Rethink. All first-degree relatives took part as single member from their family (i.e., were not related to the participating patients). Control individuals were recruited online via websites (e.g., Gumtree, Callforparticipants), and recruitment circulars at the IoPPN. The authors assert that all procedures contributing to this work comply with the ethical standards of the relevant national and institutional committees on human experimentation and with the Helsinki Declaration of 1975, as revised in 2008. All procedures were approved by the London-Harrow Research Ethics Committee [14/LO/0710].

### Material and methods

#### Demographic data

Participants completed a demographic questionnaire containing questions concerning, e.g., gender, age, nationality/ethnicity, living status and medication.

#### Experience sampling method (ESM)

Participants received an iPod with an ESM application (app) and were instructed to carry the iPod with them at all times or used their own iPhone with the app. Participants completed a short questionnaire on the device up to ten times daily when alerted by a beep. Beeps appeared pseudo-randomly between 8.00 am and 10.30 pm, for 7 consecutive days, to achieve a representative impression of a week in the participants’ lives. The ESM questionnaire contained either 30 or 34 items depending on the answer to the item ‘I am on my own’, branching into different questions when individuals were alone or not. Our main analyses focused on ‘paranoia’, ‘being in social company vs. alone’, and the ‘nature of company’.

**Fig. 1:**
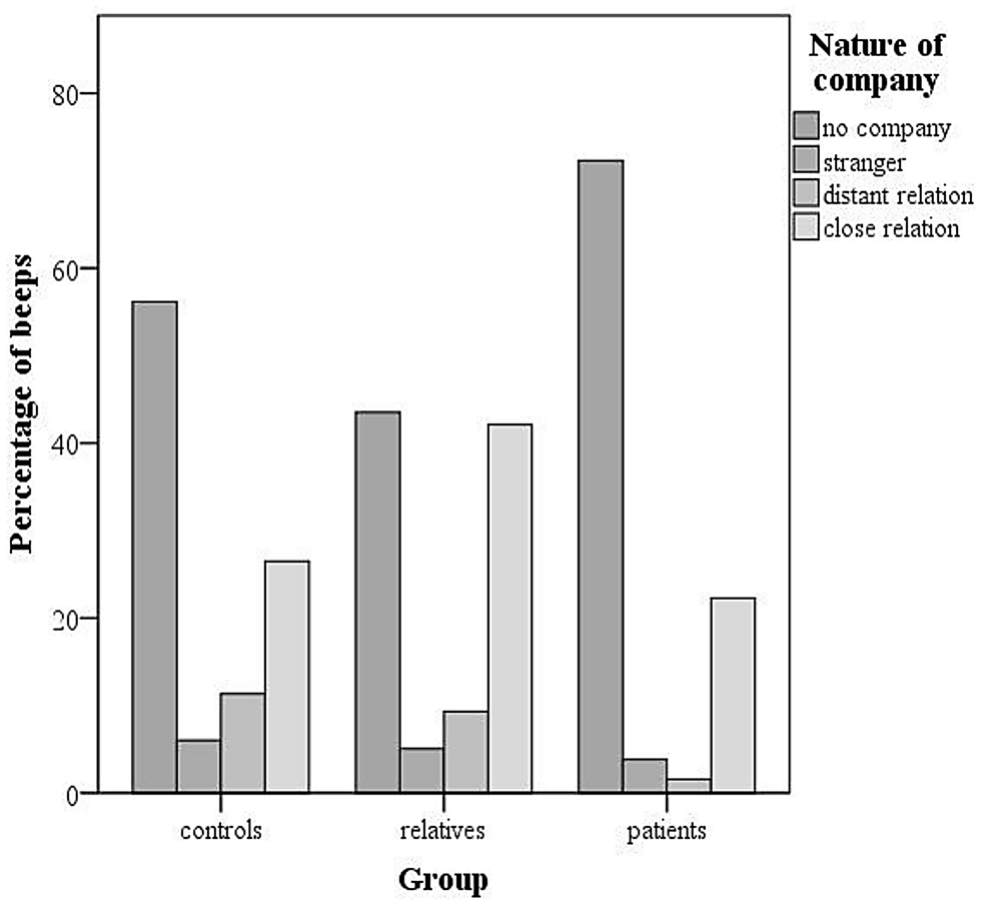
Percentage of beeps for the different company categories and groups

Paranoia was measured with an average of five items, including ‘I feel suspicious’, ‘I feel safe’ (reverse scored), ‘I feel that others …dislike me, …intend to harm me’, rated on seven-point Likert scales (1 = ‘not at all’ to 7 = ‘very’, *α* = 0.79), as previously used [9, 13]. Thewissen et al. [13] used a factor analytic approach on the raw within-participants scores and identified one factor according to the Kaiser criterion (eigenvalue > 1), which explained 75% of the total variance. Negative (< −0.84) and positive statements (> 0.80) had a strong loading on the factor and high internal consistency (Cronbach’s *α* > 0.89), which is supported by our data [13]. ESM paranoia correlated positively with the Positive and Negative Syndrome Scale Suspiciousness item (PANSS P6, *r* = 0.45, *p* < 0.01). Social company was measured by the item ‘I am on my own’ (yes/no). If this item was answered with ‘no’ the nature of company was assessed by the item ‘I am with …’, with answer options ‘partner’, ‘family’, ‘friend(s)’, ‘housemate(s)’, ‘colleague(s)’, ‘acquaintance(s)’, ‘stranger(s)’, and ‘other’. For the purpose of this study the nature of social company was divided in two categories: (1) ‘close relation’ [partner, family, friend(s)] and a combined category (2) ‘distant relation & strangers’ [housemate(s), colleague(s), acquaintance(s)] and (stranger(s), other), because patients were rarely in company of distant relations, such as colleagues (see Fig. 1). If participants indicated that they were with individuals from different categories (e.g., partner and stranger) they were grouped according to the closest person they were with. We also report positive (‘I feel relaxed,… content,… cheerful’, *α* = 0.81) and negative affect (‘I feel irritated,… low, …tense, I am ruminating’, *α* = 0.77), which have been suggested to account for the association between paranoia and social company [14].

#### Positive and Negative Syndrome Scale (PANSS)

Symptoms were assessed with the PANSS [15], which consists of a positive (7 items), negative (7 items) and general symptoms (16 items) scale, scored from 1 (absent) to 7 (extreme, see Table 1).

**Table 1:** Sample characteristics

Variable	Patients	Relatives	Controls	Group differences	*p* value for group effect
	(*n* = 29) (%)	(*n* = 20) (%)	(*n* = 26) (%)		
Gender (male)	74.9	30.3	67.1	SZC, C > R	< 0.001
Origin				SZC ≠ R ≠ C	< 0.001
British	58.7	70	75.8		
Other European	19.2	5	3.5		
African	–	15	20.7		
South American	3.9	–	–		
North American	7.7	5	–		
Asian	11.5	5	–		
Ethnicity				SZC ≠ R ≠ C	< 0.001
Black	61	25	8		
White	25	45	61		
Indian	4	15	27		
Other	13	15	4		
Education				SZC ≠ R, C	< 0.001
None/Primary	17.3	5	–		
Secondary	31	–	27		
College	34.5	30	23		
University	17.2	55	50		
Living status				SZC ≠ R, C	< 0.01
Alone	69	20	31		
Family/partner	31	60	46		
Other	–	20	23		

	*M* (SD)	*M* (SD)	*M* (SD)		
Age	39.1 (9.9)	37.27 (14.64)	36.14 (8.13)		0.60
Completed ESM beeps	54 (10.3)	45 (10.9)	51 (11.12)	SZC = R, C, R ≠ C	0.04
ESM paranoia	2.36 (1.27)	1.85 (1.01)	1.71 (0.95)	SZC ≠ R = C	0.06
PANSS P6 average (suspiciousness)	2.67 (1.22)				
PANSS positive average	1.85 (0.61)				
PANSS negative average	2.17 (0.83)				
PANSS general average	1.73 (0.36)				

*SZC* schizophrenia and other non-affective psychoses, *C* controls, *R* relatives, *ESM* experience sampling method, *PANSS* Positive and Negative Syndrome Scale

### Procedure

Testing took place at the IoPPN. Written informed consent was obtained from all participants. The study comprised two sessions. During the first, the researchers explained the study details and informed consent was taken. This study was part of a larger project and participants first completed several cognitive tasks and questionnaires, reported elsewhere (e.g., see [16]). Subsequently, those who did not own an iPhone received an iPod and all participants were given instructions to complete the app-based ESM questionnaires on this device for 7 days. Usage of the app was explained and demonstrated. After the ESM week the participants returned for the second testing session. Again, participants completed several cognitive tasks and questionnaires and the PANSS. They returned the iPod and received a £40 compensation for the study participation and reimbursement of travel costs.

### Data analysis

The data were analysed with Stata version 16. To be included in the analyses, participants had to respond validly to at least one-third of the beeps [17, 18]. Six participants (four patients, one relative and one control) were therefore removed from the dataset due to an insufficient number of completed observations. Data from one relative were not recorded due to technical problems. Data from 75 participants were included in the final analyses. Analysis of variance was used to examine group differences in age. Differences in gender composition were investigated with *χ*^2^ tests. Mixed multilevel regression analyses (XTMIXED and XTMELOGIT) were conducted to take into account the hierarchical structure of the data (i.e., multiple assessments within participants). Significant interactions and factorial main effects were tested with the CONTRAST command (Wald test, as reported in [9]). Non-significant interactions were removed from the statistical model before main effects were interpreted.

#### Group differences in time spent in social company vs. alone and paranoid thinking

First, a logistic multilevel random regression was used to assess group differences in time spent in social company vs. alone and mixed multilevel regression analysis was used to examine overall group differences in paranoia. Gender was added to the first model in a second step to investigate possible confounding of group differences in terms of time spent in social company vs. alone.

#### Relationship between time spent in social company vs. alone and paranoid thinking

To test the second hypothesis a mixed multilevel regression was run including group, being in social company vs. alone and their interaction on concurrent paranoia. The third hypothesis whether being in social company vs. alone at the previous time point (within 180 min, controlled for being in social company vs. alone at the current time point) would predict paranoia at the current time point and whether this differed between groups was investigated with lagged mixed effects multilevel regression. A time lagged logistic multilevel random regression including group, paranoia at the previous time point (within 180 min, controlled for paranoia at the current time point) and their interaction on being in social company vs. alone at the current time point was run to investigate the reverse association.

#### Relationship between the nature of social company and paranoid thinking

To test the fourth hypothesis, a logistic multilevel random regression was run with the predictors group, nature of social company (close vs. distant relation) and their interaction on concurrent paranoia. Lagged logistic multilevel random regression examined the fifth hypothesis whether the nature of social company at the previous time point (within 180 min, controlled for nature of company at the current time point) predicts current paranoia, as reported by Myin-Germeys et al. [10], and whether this relationship differed between groups.

#### Exploratory analyses

In line with previous research [14], we report exploratory analyses on the associations between being in social company vs. alone, the nature of social company and positive and negative affect.

## Results

### Sample characteristics

Sample characteristics are displayed in Table 1. The gender distribution differed significantly between the groups. Relatives differed significantly from both patients and controls (both *p*’s < 0.01), with more females in the relatives group. Patients and controls did not differ significantly (*p* = 0.56). The groups did not differ significantly in age. Patients had current primary diagnoses of schizophrenia (*n* = 23), schizoaffective disorder (*n* = 4), and psychosis not otherwise specified (*n* = 2). The majority of patients (79.3%) were not engaged with work (paid or voluntary) or study, compared to only 16.7% of controls and 21.1% of relatives. Patients’ PANSS scores are reported in Table 1.

### Group differences in time spent in social company vs. alone and paranoid thinking

On average, patients were alone at the time of 68.5%, relatives at 44.8% and controls at 56.2% of their completed beeps. Time spent alone differed significantly between the groups [*X*^2^(2) = 11.03, *p* = 0.004]. Specifically, patients had significantly higher odds of being alone than controls (OR 2.21, *p* = 0.05) and relatives (OR 4.26, *p* < 0.001), while the odds of being alone did not differ significantly between relatives and controls (OR 0.23, *p* = 0.14). Male gender was associated with significantly higher odds of being alone (OR 2.35, *p* = 0.02). When gender was added to the model the difference between patients and controls became smaller and trended towards significance (OR 1.98, *p* = 0.08). The effect for patients vs. relatives became smaller, but remained significant (OR 2.80, *p* = 0.03). The groups differed marginally significantly in their levels of paranoia [*X*^2^(2) = 5.52, *p* = 0.06]. Patients experienced marginally significantly greater paranoia than relatives [*b* = 0.47, *p* = 0.08, 95% CI (− 0.06, 1.0)] and significantly greater paranoia than controls [*b* = 0.55, *p* = 0.03, 95% CI (0.5, 1.0)], but controls and relatives did not differ significantly [*b* = 0.07, *p* = 0.78, 95% CI (− 0.47, 0.61)], see Table 1).

### Relationship between time spent in social company vs. alone and paranoid thinking

There was a significant group-by-being alone vs. in social company interaction on concurrent paranoia [*X*^2^(2) = 17.9, *p* < 0.0001]. Specifically, patients experienced significantly greater paranoia when alone than when being in social company [*b* = 0.11, *p* = 0.016, 95% CI (0.02, 0.19)]. Relatives showed the reversed pattern, with lower paranoia when alone than when being in social company (*b* = − 0.17, *p* < 0.001, 95% CI [− 0.28, − 0.07]) and in controls the association was not significant (*b* = − 0.02, *p* = 0.67, 95% CI [− 0.09, 0.06]).

Lagged analysis also showed no significant interaction of group-by-being in social company vs. alone on paranoia within 180 min later [*X*^2^(2) = 0.76, *p* = 0.68] and no significant main effect of being in social company vs. alone on paranoia within 180 min later [*X*^2^(2) = 1.46, *p* = 0.22]. The main effect of group was marginally significant [*X*^2^(2) = 5.14, *p* = 0.07]. Lagged analysis showed no significant group-by-paranoia interaction [*X*^2^(2) = 2.66, *p* = 0.27] and no main effect of paranoia at the previous time on the odds of being alone within 180 min later (OR 0.96, *p* = 0.68). The main effect of group was significant [*X*^2^(2) = 10.96, *p* = 0.004].

### Relationship between the nature of social company and paranoid thinking

The group-by-nature of company interaction on concurrent paranoia (*X*^2^(2) = 0.65, *p* = 0.72) and main effect of group were non-significant [*X*^2^(2) = 4.18, *p* = 0.12]. The main effect of nature of company on concurrent paranoia was significant [*X*^2^(2) = 4.56, *p* = 0.03]. In all groups, paranoid thinking was greater in company of strangers/distant relations than in company of close relations.

The lagged analysis showed no group-by-nature of company interaction on paranoia within 180 min later [*X*^2^(2) = 1.61, *p* = 0.44]. The main effect of group was not significant [*X*^2^(2) = 2.79, *p* = 0.25]. However, the lagged main effect of nature of company on paranoia within 180 min later was significant [*X*^2^(2) = 4.9, *p* = 0.03], showing that across all groups being with close others was associated with reduced paranoia over time.

### Exploratory analyses

There were no significant interactions between group and being in social company vs. alone and group and the nature of social company on positive affect [*X*^2^(2) = 0.98, *p* = 0.61 and *X*^2^(2) = 1, *p* = 0.60] or negative affect [*X*^2^(2) = 3.25, *p* = 0.19 and *X*^2^(2) = 0.28, *p* = 0.86]. When in social company vs. alone all groups showed significantly higher positive [*X*^2^(2) = 8.9, *p* = 0.002] and significantly lower negative affect [*X*^2^(2) = 4.14, *p* = 0.04]. There were no significant main effects of group for either positive or negative affect [*X*^2^(2) = 0.09, *p* = 0.95 and *X*^2^(2) = 0.53, *p* = 0.76, respectively]. Participants reported significantly higher positive [*X*^2^(2) = 33.2, p < 0.0001] and significantly lower negative [*X*^2^(2) = 13.4, *p* < 0.001] affect in company of close vs. strangers/distant relations. Table 2 shows averages of paranoia and affect by social company.

**Table 2:** ESM paranoia, social threat, and closeness by company category

	Alone	With others	Stranger/distant relation	Close relation
	*M* (SD)	*M* (SD)	*M* (SD)	*M* (SD)
Paranoia				
Control	1.86 (1.03)	1.52 (1.85)	1.55 (0.70)	1.49 (0.77)
Relative	1.77 (0.86)	1.92 (1.11)	2.16 (1.13)	1.82 (1.09)
Patient	2.56 (1.25)	1.92 (1.20)	2.22 (1.24)	1.85 (1.18)
Positive affect				
Control	4.65 (0.89)	4.94 (0.98)	4.71 (0.87)	5.10 (1.01)
Relative	4.81 (1.43)	4.75 (1.42)	4.48 (1.44)	4.87 (1.39)
Patient	4.83 (1.19)	4.86 (1.19)	4.64 (1.30)	4.91 (1.17)
Negative affect				
Control	2.38 (1.11)	2.18 (1.06)	2.02 (0.93)	2.28 (1.11)
Relative	2.59 (1.40)	2.63 (1.45)	2.99 (1.41)	2.47 (1.44)
Patient	2.57 (1.27)	2.34 (1.17)	2.59 (1.34)	2.28 (1.12)

All items are rated on a 1–7 Likert scale

## Discussion

The present study is the first to investigate the associations between real-life social contact and paranoid thinking across the psychosis continuum. Our findings show that patients are most frequently alone and that being alone, compared to being with others, is associated with higher levels of concurrent paranoid thinking specifically in patients. Across all groups, social contact with familiar people was associated with lower paranoid thinking than being with less familiar people or strangers. These findings highlight the importance of social contact for mental wellbeing and relief from symptoms.

### Time spent in social company vs. alone and paranoid thinking

In line with extensive literature on social functioning in individuals with psychosis our findings showed that patients were alone significantly more often than first-degree relatives and controls [1, 14, 19, 20]. This effect was partly explained by gender, with males being alone more frequently. It has been hypothesized that social withdrawal could reflect a safety behaviour in which patients avoid company because they see others as threatening and assume that social withdrawal helps them to cope with the threat. Importantly, in reality this safety behaviour does the opposite, i.e., the threat is not disconfirmed, therefore paranoid thinking is maintained or even aggravated. The current data partially supported the idea of social withdrawal as safety behaviour. The time-lagged analysis showed that levels of paranoid thinking at a previous time point did not predict future social isolation (i.e., being alone vs. not) and social isolation also did not predict paranoia over time (within 180 min later). However, being in the social company of others as compared to being alone was actually associated with lower levels of paranoia at the same time. This suggests that social interactions may disconfirm paranoid thought and/or provide distraction from negative paranoid thoughts. Surprisingly, relatives demonstrated the reverse pattern–namely, less paranoid thinking when alone compared to when being in company of others. Despite the differential associations between social company and paranoia, being in social company was associated with higher positive and lower negative affect in all three groups as compared to being alone, in line with previous reports [14]. It is possible that while during social interaction affect is generally improved in relatives, social interaction could also increase social stress or anxiety, which could foster paranoia in those with an elevated familial-risk of psychosis (who do not receive anti-psychotic medication).

Our findings suggest that patients’ tendency to be alone frequently is not entirely driven by safety behaviours, but that it may be due to other factors, such as for instance the lack of opportunity for social interaction through leisure or work. The majority of patients (79.3%) were not engaged with work (paid or voluntary) or study, compared to only 16.7% of the controls and 21.1% of the relatives. Also, individuals in the patient group were more than twice as likely to live alone compared to relatives and controls. Finally, less social contact could be the consequence of other factors that have been linked to a diagnosis of non-affective psychosis, such as poor social (cognitive) skills and theory of mind [21, 22], difficulties to establish trust [23, 24], or social stigma [25].

### Relationship between the nature of social company and paranoid thinking

The current data did support our hypothesis about the sensitivity to the nature of social company, i.e., social context or familiarity, in individuals with a diagnosis of non-affective psychosis. Similar to relatives and controls, patients reported more paranoia when they were in company of strangers or less familiar others, compared to familiar others. This pattern seems adaptive, given that one has to be more cautious of the intentions of less familiar people. In line with Myin-Germeys et al. [10], our results also showed that being with close others predicted reduced paranoid thinking at the next momentary assessment (within 180 min), suggesting a protective nature of being in social company, particularly of familiar others, against paranoid thinking. The fact that the current findings contrast previous research in individuals with high (but not medium or low) trait paranoia, which suggested that paranoia during social interactions becomes independent of the nature of the social relationship [9], highlights methodological considerations. In the current study, participants with a diagnosis of non-affective psychosis were in fairly stable stages of the disorder (similar to the sample described in [10]). Thus, our findings may not generalize to trait paranoia or the more acute paranoid stages of clinical psychosis. Future studies could usefully investigate the relationship of social interaction and paranoid symptomology as illness severity changes during treatment.

### Conclusion

Social isolation is often chronic in individuals with a psychotic disorder [1] and has been associated with lower quality of life and a range of negative health outcomes [26]. Our data highlight that social isolation is common in individuals with a diagnosis of non-affective psychosis. The large amount of time that individuals with psychosis spent alone is especially important given their elevated levels of paranoia when being alone. These findings highlight the importance of psychological interventions that stimulate social engagement and reduce social stigma, such as Cognitive Behavioural Therapy for Psychosis, social (cognitive) skill and integration training and psycho-education for individuals with psychosis and the need for attention to their socio-economic environment to optimize patients’ opportunities for social interaction [27–29]. Recent developments in digital technology may enable interpersonal contact. However, further research on how such methods can be leveraged to reduce social isolation in patients is needed (e.g., [30]). In many cases where improvements in social contact are not immediately possible in everyday life, therapy settings could be used to establish low threshold social contact (e.g., through social prescribing, participation in support groups or buddying schemes) [31, 32] and to teach patients to strengthen their self-efficacy and well-being in periods where they are alone [33].

## Data Availability

The data that support the findings of this study are available from the corresponding author, AKJF, upon reasonable request.
